# Population Pharmacokinetic Study of Benznidazole in Pediatric Chagas Disease Suggests Efficacy despite Lower Plasma Concentrations than in Adults

**DOI:** 10.1371/journal.pntd.0002907

**Published:** 2014-05-22

**Authors:** Jaime Altcheh, Guillermo Moscatelli, Guido Mastrantonio, Samanta Moroni, Norberto Giglio, Maria Elena Marson, Griselda Ballering, Margarita Bisio, Gideon Koren, Facundo García-Bournissen

**Affiliations:** 1 Servicio de Parasitología y Chagas, Hospital de Niños Ricardo Gutiérrez, Ciudad de Buenos Aires, Argentina; 2 Área de Toxicología, Departamento de Ciencias Biológicas, Facultad de Ciencias Exactas, Universidad Nacional de La Plata, La Plata, Provincia de Buenos Aires, Argentina; 3 Division of Clinical Pharmacology & Toxicology, Hospital for Sick Children, University of Toronto, Toronto, Ontario, Canada; Universidad Autónoma de Yucatán, Mexico

## Abstract

**Introduction:**

Chagas disease, caused by the parasite *Trypanosoma cruzi*, can lead to long term cardiac morbidity. Treatment of children with benznidazole is effective, but no pediatric pharmacokinetics data are available and clinical pharmacology information on the drug is scarce.

**Patients and Methods:**

Prospective population pharmacokinetic (PK) cohort study in children 2–12 years old with Chagas disease treated with oral benznidazole 5–8 mg/kg/day BID for 60 days. (clinicaltrials.gov #NCT00699387).

**Results:**

Forty children were enrolled in the study. Mean age was 7.3 years. A total of 117 samples were obtained from 38 patients for PK analysis. A one compartment model best fit the data. Weight-corrected clearance rate (CL/F) showed a good correlation with age, with younger patients having a significantly higher CL/F than older children and adults. Simulated median steady-state benznidazole concentrations, based on model parameters, were lower for children in our study than for adults and lowest for children under 7 years of age. Treatment was efficacious in the 37 patients who completed the treatment course, and well tolerated, with few, and mild, adverse drug reactions (ADRs).

**Discussion:**

Observed benznidazole plasma concentrations in children were markedly lower than those previously reported in adults (treated with comparable mg/kg doses), possibly due to a higher CL/F in smaller children. These lower blood concentrations were nevertheless associated to a high therapeutic response in our cohort. Unlike adults, children have few adverse reactions to the drug, suggesting that there may be a direct correlation between drug concentrations and incidence of ADRs. Our results suggest that studies with lower doses in adults may be warranted.

**Trial Registration:**

ClinicalTrails.gov NCT00699387

## Author Summary

Chagas disease is a parasitic disease endemic to the Americas. Long term complications include severe cardiac involvement, which can lead to severe disability and death of affected individuals. Treatment options for Chagas disease are limited to 2 drugs (benznidazole and nifurtimox), but knowledge of the pharmacology of these drugs is lacking, particularly in childhood. To date, no pharmacokinetics data are available in children. We conducted the first population pharmacokinetic study of benznidazole in children. Interestingly, we found that elimination of the drug is significantly faster in children than in adults, leading to lower plasma concentrations. However, unlike adults, all children in the study responded well and had few adverse reactions to the drug. These findings suggest that adjustment of current adult doses may be beneficial.

## Introduction

Chagas disease (ChD) is a parasitic infection caused by *Trypanosoma cruzi*.[1] Approximately 15 million people are affected with ChD in Latin America, with 10,000 annual deaths due to complications.[1], [2] Infection occurs most commonly in children, by the vectorial or congenital route. Left untreated, ChD leads to cardiac and/or gastrointestinal morbidity and mortality years to decades later.[1], [3] ChD is endemic in the Americas, including the US, but infected patients can also be found in Europe, Australia, Japan and other non-endemic countries due to migration.[4], [5], [3], [6]

Only two medications are currently available for the treatment of ChD, benznidazole and nifurtimox.[7], [8] Even though both drugs were developed over 4 decades ago, there is little information available on their clinical pharmacology, particularly for special populations such as children.[8], [9]

Based on a small number of studies, treatment of pediatric ChD with benznidazole is considered to be effective and well tolerated, with observed response rates nearing 90% in some series.[10], [8], [9], [11], [12], [13] However, treatment schedules are based on limited data, mostly coming from the only two pharmacokinetics (PK) studies conducted, with a limited number of adult patients.[14], [15] No information on benznidazole PK is available for the pediatric population.[9], [16] This lack of important data may lead to significant risks for children, a particularly vulnerable population.

Given this knowledge void, we have conducted the first pediatric population PK study of benznidazole in a cohort of children with ChD.

## Methods

We enrolled children between 2 and 12 years of age with ChD between April 2008 and November 2010 at the Parasitology and Chagas Service, Buenos Aires Children's Hospital “R Gutierrez”, Argentina.

Subjects with *T. cruzi* infection, defined as a positive result in at least 2 distinct serologic tests (ELISA, Wiener Laboratory, Rosario, Argentina; indirect hemagglutination, Polychaco Laboratory, Buenos Aires, Argentina; or particle agglutination, Fujirebio, Tokyo, Japan), and no previous treatment for ChD were considered for inclusion. Parasitemia was evaluated by real time polymerase chain reaction (qPCR) for parasite nuclear satellite DNA in blood. [17], [18], [19] Exclusion criteria were: pregnancy (pregnancy tests were performed for all adolescent girls before inclusion); treatment with any investigational drug in the month before enrollment; cardiovascular, hepatic, neurologic, endocrine, or other major systemic diseases; and immunocompromised patients; Not being able to comply with the study, or to provide consent from a guardian and assent by the child.

Patients were treated with benznidazole (Radanil, Roche, São Paulo, Brazil) 100 mg tablets, dose: 5-8 mg/kg/d bid p.o. for 60 days.[20], [12] Tablets were fractioned by a hospital pharmacist in an individualized manner, and re-packaged preparations with the individual doses were provided to each patient, accompanied by written indications for administration. Medication was provided in monthly batches, and adherence was assessed by tablet counting at each visit. Caregivers also completed a treatment diary to record doses administered, times of doses, symptoms, and problems associated to the treatment. The diary was reviewed in every clinic visit. All patients were given a phone number to contact the researchers if they had any questions or issues with the treatment, and were invited back to clinic in any occasion if there were doubts or concerns.

A detailed clinical history, physical examination, and routine laboratory tests[20], [21] were performed at diagnosis and 7, 30, and 60 days after start of treatment. Signs and symptoms suggesting ADRs were specifically inquired for and recorded during each hospital visit. Serologic tests for detection of antibodies against *T. cruzi* were done before and at 30 and 60 days of pharmacotherapy, and every 3–6 months after treatment. Treatment response was evaluated by *T. cruzi* specific qPCR at the end of treatment.[17], [18], [19] Cardiological evaluation, including echocardiogram and electrocardiogram, was conducted before the start of the treatment and yearly afterwards, as per current clinical, and Chagas Service, guidelines applied to all ChD patients treated in our Hospital.

### Ethics statement

The study was approved by the Ethics and Research Review Boards, Buenos Aires Children's Hospital “R Gutierrez”, and the Argentine National Drug and Food Administration (ANMAT), Ministry of Health, Argentina. Written informed consent was required from patients' legal representatives, as well as assent from the patient when appropriate. The study was registered in clinicaltrials.gov (#NCT00699387).

### Measurement of benznidazole in plasma samples

#### Samples for population PK analysis

At least 3 blood samples per child were obtained at random times within pre-specified windows. The sampling windows were as follows: for patients taking the first dose of the drug, the three sampling windows were: 1) 0–2 hs 2) 2–6 hs and 3) 6–12 hs post dose; for patients at the steady state phase (i.e. at least after 3 days of treatment; days 3–59 of treatment), the three sampling windows were: 1) trough (i.e. before the following dose); 2) 0–2 hs and 3) 2–6 hs post dose; finally, for patients receiving the last dose of benznidazole, the three sampling windows: 1) 12–18 h; 2) 18–24 h and 3) 24–36 h post dose.

Whenever possible, blood samples were taken through an IV catheter. Thirty four patients provided 3 samples, 2 patients provided 5 samples, 1 patient provided 4 samples and 1 patient provided 1 sample. In the three patients with more than 3 samples, the extra samples were opportunistically obtained from left over blood taken for routine laboratory tests. After extraction, blood was immediately spinned, and plasma separated, lyophilized and stored at −4°C until analysis.

Benznidazole was measured by HPLC. Briefly, 2 mL of ethyl acetate were added to each 1 mL of lyophilized plasma sample. The mixture was manually shaken and precipitated with trichloroacetic acid (30% v/v), vortexed for one minute and sonicated for 5 minutes. The mixture was then centrifuged at 8000 g for 10 min, the supernatant put into a round bottom flask and roto-evaporated to dryness. The residue was re-suspended in 600 µL of the chromatographic mobile phase and injected into the HPLC system. HPLC analysis was performed by isocratic elution with a flow rate of 1.0 ml/min with UV detection at 313 nm. The mobile phase composition was glycine buffer/acetonitrile (75∶25 v/v). The glycine buffer was an aqueous solution of glycine 0.20 M and sodium octanesulphonate 5.0 mM at pH 2.5. The limit of detection (LOD) and limit of quantitation (LOQ) were 0.14 mg/L, 0.32 mg/L respectively. Interday accuracy was 6.3% and precision 3.4%. The method was linear up to 20.00 mg/L.[22], [23]

### Adult data

Adult data was obtained from the original benznidazole studies by Raaflaub et al.[14], [15], which contain tables with individual blood concentrations of benznidazole after single dose[14] and multiple dose (30 days treatment) treatments [15], in healthy volunteers (N = 6, all female) and adult ChD patients (N = 8, 50% female), respectively. Mean weight of the individuals in these studies was 55.4 kg (sd = 7.8), and mean age was 33.3 years (sd = 12).

### Population PK (POPPK) analysis

#### POPPK modeling

Population PK parameters were calculated using data obtained from the children in our study, as well as published adult data.[14], [15] Adult data was added to obtain a more comprehensive model that would encompass as wide an age range as possible.

#### Structural model

POPPK modeling was carried out using nonlinear mixed-effects modeling as implemented with NONMEM VII (version 7.0; ICON Development Solutions, Ellicott City, MD). NONMEM uses mixed (fixed and random)-effects nonlinear regression to estimate average population parameters and inter- and intraindividual (i.e., residual) variability. A stepwise procedure was used to find the model that offered the best fit for the data. Classic one- and two-compartment models were evaluated.

#### Covariate model

The influence of covariates was assessed first by graphical visual inspection of PK parameters vs. the covariates plots. Potential or known influential covariates were incorporated sequentially into the PK model. The typical value of a given parameter was modeled to depend linearly on each tested covariate. Covariates tested included body weight, age, dose, gender and whether the subject was a child or adult (i.e. data coming from our study population or the literature). An allometric approach of weight on clearance (CL/F), both by using the fixed exponential 0.75, and by allowing NONMEM to estimate the coefficient, was also tested.

#### Error models

Inter-individual variations in PK parameters were described using exponential error models. A proportional error model was used to describe the intrapatient (residual) variability.

#### Parameter estimation and model selection

Data were fitted using a combined sequential strategy. First a Monte Carlo importance sampling method (IMP) was used to initially estimate parameters, followed by confirmation with the Markov chain Monte Carlo stochastic approximation expectation maximization (SAEM) method, as implemented in NONMEM VII with the ADVAN5 subroutine. Model selection was based on the likelihood ratio test, PK parameter point estimates and their respective confidence intervals (CI), goodness-of-fit plots, and visual predictive checks. Finally, a model was regarded as a statistically significant improvement over a previous model if it produced a decrease in objective function (ΔOF) of >3.84 for one additional parameter (χ^2^; P<0.05).

#### Model evaluation

The final selected model was validated on the basis of the standard error of the estimates and visual predictive check (VPC) plot. The bootstrap method with replacement was used to assess stability of the final model and to construct the CIs of the PK parameters, using PsN-Toolkit version 3.5.3. One thousand data sets were reconstructed by resampling from the original data. Mean and 95% CI values of the parameter estimates for each of the replicate data sets were compared with those from the original data.

#### Simulation

Individual benznidazole steady-state concentrations (Css) were derived from the classical Css equation:

Css = F× (Dose×WT.ind)/(CL.ind×tau)

where F is the bioavailability of orally administered benznidazole (set at 100% for simplicity), Dose is the daily benznidazole dose, set at 7 mg/kg/day, WT.ind is the individual patient weight, CL.ind is the estimated individual (Bayesian) benznidazole clearance rate (in L/day) as per POPPK analysis, and tau is the dosing interval (set to 1 in this case, as the dose is expressed in mg/day).

All statistical calculations were performed in R statistical language, V.2.14.1 R Foundation for Statistical Computing, Vienna, Austria, (www.R-project.org). Diagnostic graphs were produced in R, with the package Xpose 4.[24]

## Results

### Demographics

**Figure 1:**
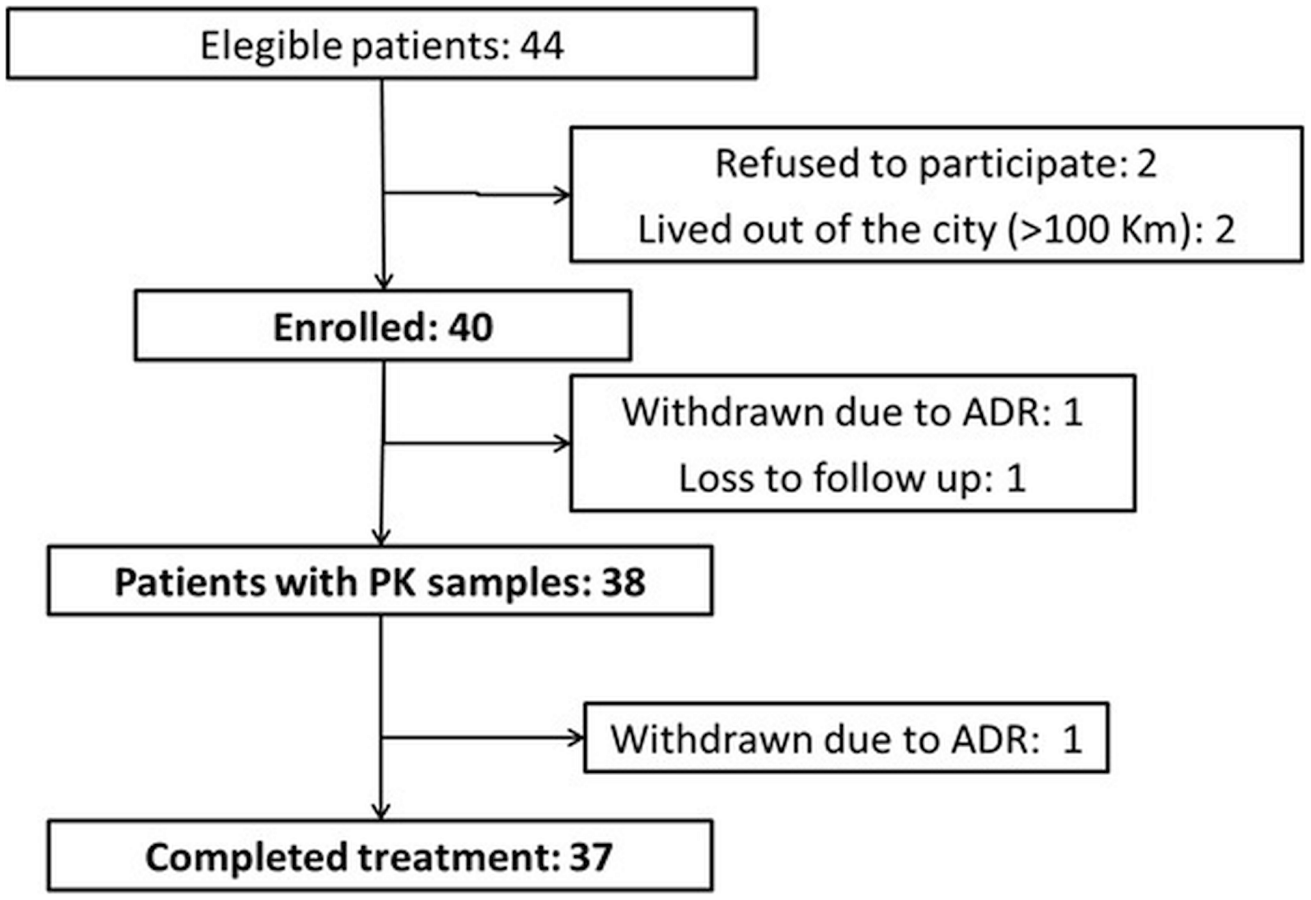
Study flowchart.

Forty children diagnosed with ChD were enrolled in the study (Figure 1). Thirty eight patients contributed 117 samples for PK analysis and 37 (93%) completed 60 days of treatment (one patient contributed PK samples before withdrawing due to an adverse drug reaction). Most patients (90%) resided in the city of Buenos Aires or the greater Buenos Aires area, a non-endemic area for vector transmission; the remaining patients (10%) were referred from rural areas. Route of infection was congenital in 55% of the patients, vectorial in 5% and undefined in 40%. Mean age was 7.3 years (SD: 3.5). Eighteen patients were girls (45%). Mean weight was 27.2 kg (SD 12.8; range 11.5–64.0 kg). No subjects received chronic concomitant medications. All patients were asymptomatic with no cardiac involvement or other ChD–associated pathology, and no comorbidities or laboratory abnormalities, at enrollment.

At diagnosis, qPCR was positive in 31/37 (84%) patients for which samples were available. No initial samples were available for qPCR in the remaining 3 enrolled patients due to difficult blood extraction. Mean benznidazole dose was 6.4 mg/kg/day (SD 1; range 5.0–8.7) in two divided daily doses. Good adherence was observed based on tablet count and treatment diary review.

Treatment response was high, with all 37 patients who completed 60 days of treatment having negative qPCR at the end of treatment (day 60). All patients were followed for at least 1.5 years after treatment completion and a steady decrease of specific *T. cruzi* antibodies and persistently negative qPCR was observed. Also, qPCR was negative in all 21 patients for which samples were available after 2 years of treatment. No cardiac involvement and no long-term adverse consequences of treatment have been observed in any of the patients. Most of the patients still had positive antibodies at 2 years follow up (albeit at lower titers than before treatment), a finding consistent with previous observations in this age group and in adults. This confirms that antibody tests require much longer periods of follow up for negative results to be observed, unlike disappearance of parasitemia which commonly occurs early after treatment.

Four (10%) patients had adverse drug reactions (ADRs) related to benznidazole, all mild (1 mild rash, 1 moderate prurigo, 1 generalized rash without systemic involvement, and 1 moderate eosinophilia). Mean age of children with ADRs was 8.6 years. Three out of four children with ADRs were over 7 years old. All ADRs subsided with symptomatic treatment (antihistamines) and temporary drug discontinuation, and all patients recovered uneventfully. In 2 cases rash reappeared with drug reintroduction, requiring patient withdrawal from the study (Figure 1). These 2 patients were successfully treated with nifurtimox later on, with good response. One further patient withdrew from the study due to maternal decision.

### Population PK analysis

Samples for population PK analysis were obtained from 38 patients (95%) in our cohort, and 14 adult patients from the literature.[14], [15] For 2 patients in our cohort, samples could not be obtained due to early withdrawal from the study. The total number of samples for PK analysis from our cohort was 117, and 168 benznidazole plasma concentrations were obtained from published adult data.[14], [15]

#### Initial POPPK analysis (no covariates)

A one-compartment model with first-order absorption best described the benznidazole data; no significant statistical improvement was obtained by using a two-compartment model, zero-order absorption or other models (i.e. ΔOF<3.8). A proportional error model best described residual variability.

POPPK parameters and their variability are listed in Table 1. Briefly, Ka was 0.638 h^−1^, V/F 23.2 L and CL/F 1.54 L/h. Internal validity of the final model was also confirmed using bootstrap-generated data sets. Goodness-of-fit diagnostic plots results suggested acceptable model performance and visual predicted check (VPC) plots for drug concentrations in plasma showed conformity of model prediction with the observed data (data not shown).

**Table 1:** POPPK parameter estimates.

Parameter	Median [ 95% CI]	Interindividual variability [ 95% CI]
Initial model (no covariates)		
**Ka (h^−1^)**	0.638 h^−1^ [0.34;1.52]	123% [48.9;300%]
**V/F (L)**	23.2 L [18.23;42.6]	74.2% [35.2;168.2%]
**CL/F (L/h)**	1.54 L/h [1.29;1.91]	61.6% [33.5;90.3%]
**Residual error (Proportional)**	27% [21;33.9]	

Simple one-compartment model without o covariates.

Individual point estimates for CL/F were significantly lower in children in our study (<13 years old) than for adults (median 1.37 and 1.7 L/h respectively; p<0.01). Similarly, individual point estimates for V/F were significantly different between children and adults (median 22.98 and 32.56 L/h respectively; p<0.01). Estimates for Ka did not differ among groups.

#### Covariate analysis

Stepwise covariate analysis identified patients' weight to have a statistically significant influence on V/F (ΔOF −16.34; p<0.001). Other covariates such as being a child or an adult had statistically significant influence on V/F in univariate analysis. However, this influence was not confirmed when added to a multivariate model with weight, and these covariates were therefore excluded from the final analysis. Addition of gender to the model did not improve the fit of the data when tested on either parameter (V/F, Ka or CL/F) in the final model. Covariate relationships other than linear (e.g. exponential, and sigmoidal), were evaluated but did not lead to any improvement in the fit of the model. Also, visual inspection of the data did not support any specific non-linear relationship for most covariates. We specifically attempted to fit a sigmoidal relationship between CL/F and Weight, CL/F and Age, and V/F and Weight, but the results were disappointing.

Additional modeling, including weight as a covariate simultaneously on V/F and CL/F led to further improvement in the OF (ΔOF −48.65; p<0.001)(Table 2). Addition of Age as a covariate for CL/F also improved the OF (ΔOF −14.77; p<0.001).

**Table 2:** POPPK parameter estimates.

Parameter	Median [ 95% CI]	Interindividual variability [ 95% CI]
Final model		
**Ka (h^−1^)**	0.294 h^−1^ [0.17;0.606]	246% [99;258]
**V/F (L) b0+b1×WT**	b0 = −5.73 [−8.106; −2.999] b1 = 0.707 [0.632;0.802]	27.05% [5;17%]
**CL/F (L/h) b0+b1×AGE+ (WT/70)∧b2**	b0 = 0.418 [0;1.654] b1 = 0.0049 [0;0.0186] b2 = 0.0743 [0;3.497]	43.35% [34;100%]
**Residual error (Proportional)**	27% [22.8;32.3%]	

One-compartment model with covariates (weight on volume, and age and weight on clearance).

Final parameter estimates were as follows:

V/F = −5.73+0.707× Weight (kg)

CL/F = 0.418+0.0049× Age (ys) + (Weight/70)^0.0743^

**Figure 2:**
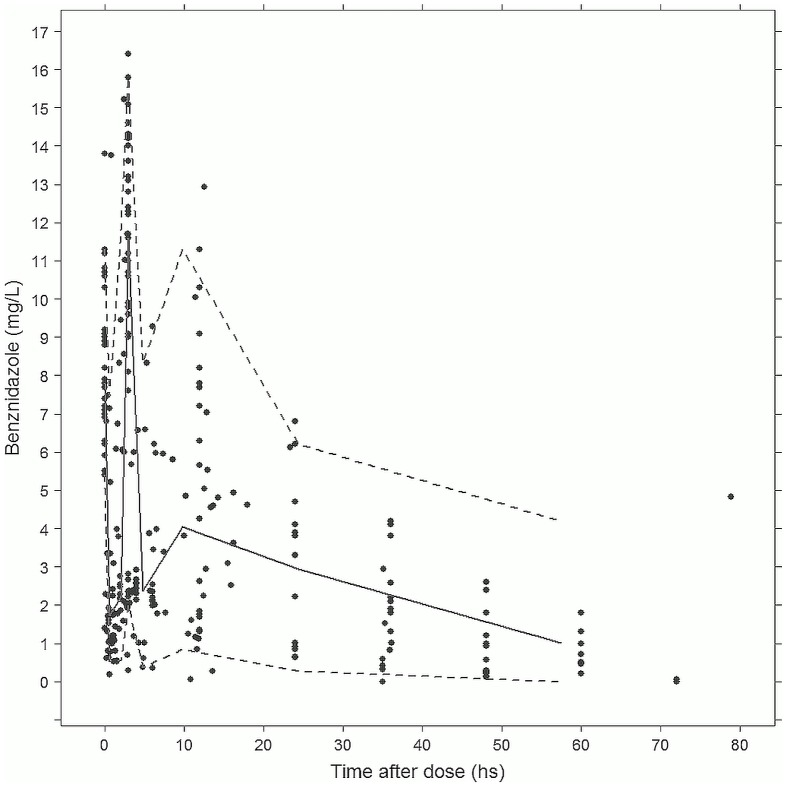
VPC plot for benznidazole concentrations in plasma vs time after each dose – final model (Covariates: Weight on V, Weight on CL, Age on CL). (solid line: median, discontinuous lines: 5 to 95% prediction interval).

**Figure 3:**
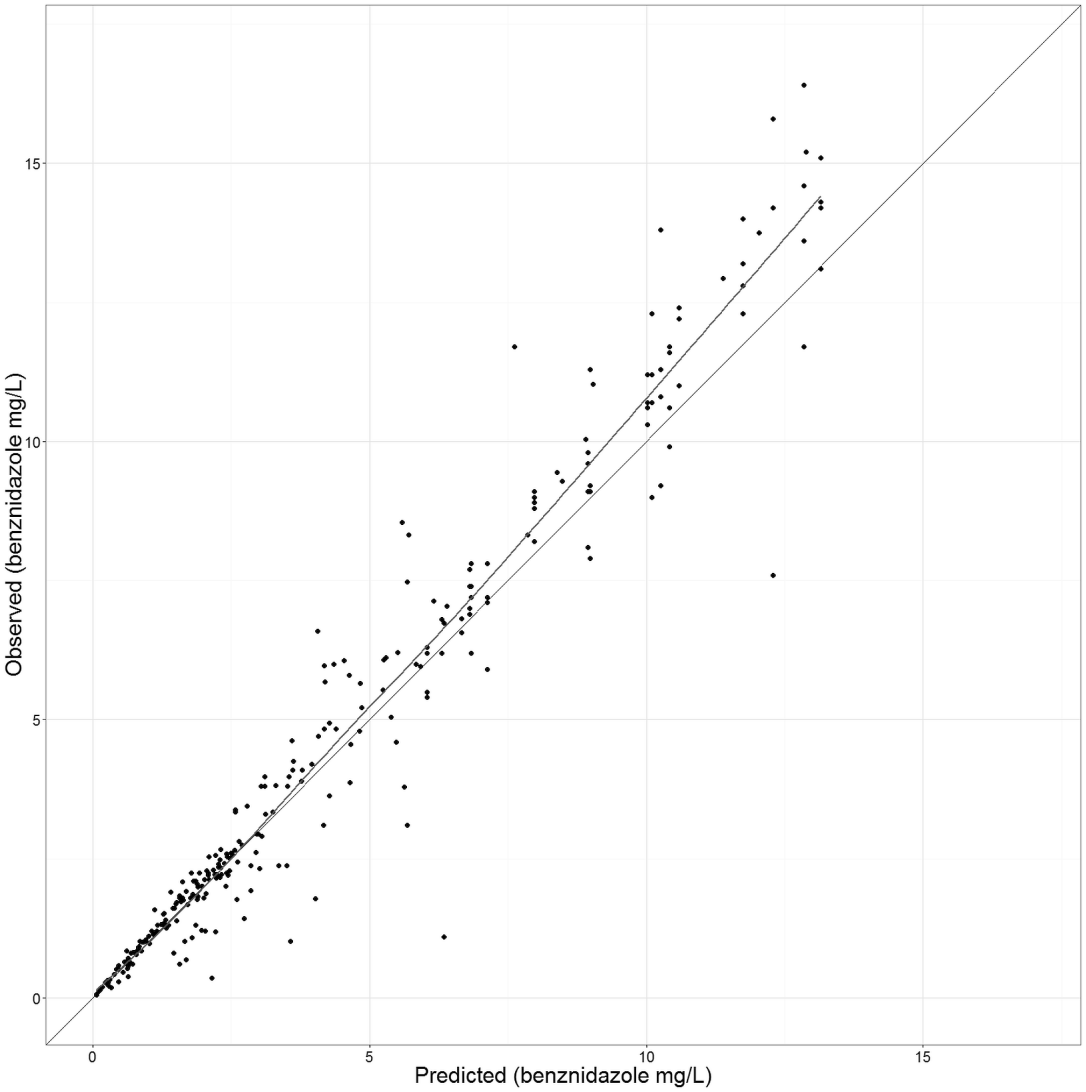
Goodness of fit plot, final model. Individual predictions vs observed data (benznidazole in mg/L).

Final parameters estimates, with 95% CI, can be found in Table 2. VPC plots for drug concentrations in plasma showed conformity of final model prediction with the observed data (Figure 2). Internal validity of the final model was also confirmed using the bootstrap-generated data sets (Table 2). Goodness-of-fit diagnostic plots results (Figure 3) suggested acceptable model performance.

**Figure 4:**
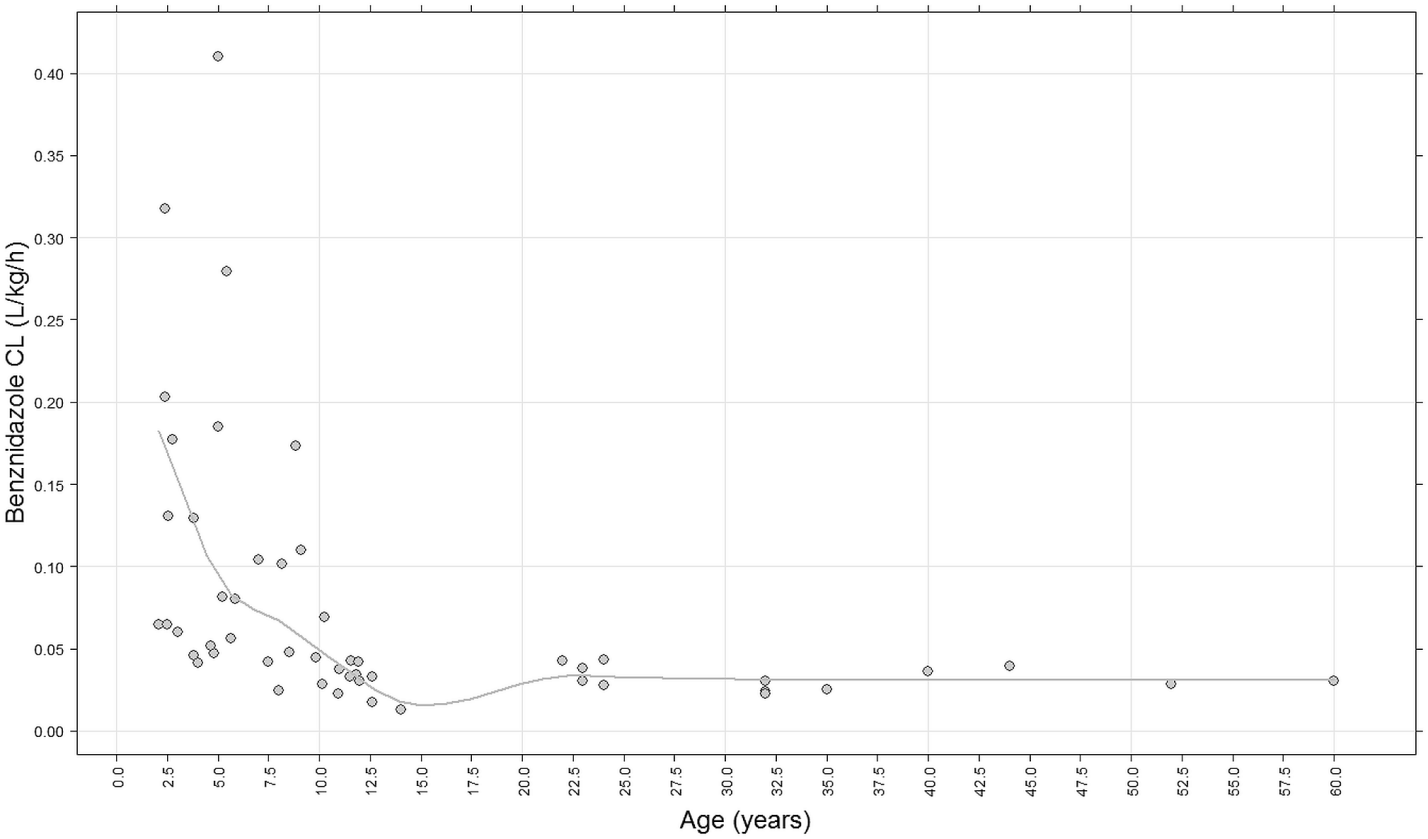
Individual predicted CL/F Data for patients over 12 years old were obtained from the literature. [14], [15]

Individual weight-corrected estimates for CL/F were significantly higher for children than for adults (median clearance 0.0615 L/Kg/h vs. 0.0301 L/Kg/h respectively; p<0.01). Children under 7 years old had the highest weight-corrected CL/F compared to older children and adults (Table 3). Individual weight-corrected CL/F vs age are depicted in Figure 4.

**Table 3:** Weight-corrected CL/F by age group.

Age	2-7 years old	7-12 years old	Adults
**CL/F (L/Kg/hr)**	0.0806	0.0424	0.0301
**(Median [95% CI])**	[0.058-0.177]	[0.034-0.069]	[0.027-0.033]

POPPK parameter–based simulation yielded a global (children and adults) median Css of 5.81 mg/L, with children in our study having a median simulated Css of 4.53 mg/L (95% CI for the median: 3.73–5.58) compared to a median Css for adults of 10.96 mg/L (95% CI for the median: 7.74–15.43). Particularly, children under 7 years of age had lower Css (Median Css 3.61 mg/L) compared to children over 7 years old (Median Css 6.88 mg/L) and adults (Table 4).

**Table 4:** Simulated Css (1000 simulations; dose 7 mg/kg/day).

Age	2-7 years old	7-12 years old	Adults
**Css (mg/L)**	3.61	6.88	9.69
**(Median [95% CI])**	[1.61; 4.87]	[3.54; 8.37]	[8.45; 10.42]
**Half life (hours)**	3.04	9.41	12.77
**(Median [95% CI])**	[1.89; 3.45]	[6.96; 11.59]	[10.84; 14.28]

## Discussion

Acute ChD occurs mainly in children, either by congenital or vector transmission. The acute infection is mostly asymptomatic and gives way to a chronic, mostly silent, phase that can last decades. [25], [2], [1] During this latter phase, internal organs may be progressively affected, leading to cardiac involvement in approximately 30% of patients. [26] In spite of the large potential public health benefit of treating pediatric ChD cases, no new treatments have been introduced for this population in decades, and the pharmacology of the only two drugs available (i.e. benznidazole and nifurtimox) was never studied in children.[8] Even though treatment of children with ChD with benznidazole has been shown to be safe and effective,[13], [11], [8], [27], [28], [29] lack of pediatric PK data makes dosing decisions a matter of guesswork. In fact, currently used pediatric dosing guidelines were developed by merely applying adult dosing to children (using weight-based adjustments).

Benznidazole population PK modeling in our study shows that weight-corrected CL/F is well correlated with age, with younger patients having a significantly higher CL/F than older children and adults. This would result in shorter half-lives (i.e. faster elimination) and in lower accumulation in smaller children compared to older children and adults, in the current therapeutic context where patients are treated with weight-adjusted doses (e.g. 5–8 mg/kg/day of benznidazole).

Many reasons can explain the observed dependency of CL/F on age. In particular, it is well-known that weight-corrected CL/F in children 2–6 years old is significantly higher than in older children and adults for many drugs, cleared by both the kidney or the liver.[30], [31], [32] Unfortunately, to date no information is available on the specific metabolic pathways involved in the elimination of benznidazole, which hinders further speculation on factors that may potentially affect its elimination, such as interactions with other drugs or food. [33] It is possible that the observed age-dependent variability in CL/F is a function of increasing bioavailability (F) across ages, rather than larger clearance rate at early ages. Unfortunately, this hypothesis is impossible to rule out at the present time due to the lack of an intravenous benznidazole formulation. However, this possibility seems unlikely in light of the high lipophilicity of the drug, and animal data suggesting complete oral absorption in mice and dogs, and due to the fact that V/F should also be affected.[34]

Unlike children, treatment of adults with benznidazole is associated to a significant incidence of ADRs,[11], [35], [32] including severe ADRs [32], [10], [27], [21], [11], [13]. Benznidazole-associated ADRs are a frequent reason for lack of treatment adherence, and possibly treatment failure, in adults.[11], [32] The noted [10], [11], [13] inverse correlation between ADRs incidence and age was also suggested by the data in our study, with only 1 out of 4 patients with ADRs under 7 years of age.

Mechanisms responsible for the observed age-dependent differences in incidence of ADRs are not clear. No studies to date have formally evaluated lower daily doses of benznidazole in adults. In an observational study, Pinazo et al. [36] failed to find a correlation between benznidazole trough levels and the incidence of ADRs in a population of 54 adult patients with ChD. Unfortunately, the PK data from this study was limited to trough levels which may vary widely depending on time of intake of the previous dose. Also, observed differences in drug concentrations among adult patients were in general much smaller than those between adults and children in our study.

We observed markedly lower benznidazole plasma concentrations in children than those previously reported in adults (treated with comparable mg/kg doses). In spite of these lower concentrations, treatment in children was effective and well tolerated, with a high response rate (as evaluated by negative qPCR at the end of treatment) and few ADRs. Based on our results (Figure 4), we expect that children older than 7 years old, including teenagers, would behave more similarly to adults than to younger children from the point of view of PK and pharmacodynamics of the drug (e.g. incidence of ADRs). It is also conceivable that benznidazole dose reduction for adults, to obtain systemic exposures similar to those in children, would not have a detrimental impact on treatment response, but may still lead to a reduction in incidence of ADRs or at least to the development of drug sparing strategies.

Our study has some limitations that should be kept in mind when interpreting the results. The population studied was restricted to children 2–12 years old, making these results difficult to extrapolate to younger patients. A population PK study of benznidazole enrolling patients younger than 2 years of age is currently in its final stages in our centers (clinicaltrials.gov # NCT01549236) to address this limitation. Also, adult data included in the analysis came from a small number of subjects for which data was publicly available [14], [15]. These subjects were either healthy volunteers or relatively young and healthy chronic ChD patients (i.e. without organ involvement). Therefore, extrapolation of the results to other adults, particularly sicker ones, should be done with care (and, ideally, within the context of a clinical trial).

### Conclusions

Observed benznidazole concentrations in children were markedly lower than those reported in adults (treated with comparable mg/kg doses). In spite of these lower concentrations, treatment was effective and well tolerated, with few ADRs, a marked difference from adults. If confirmed, our results would suggest that further studies to evaluate dosing modifications in adults may be beneficial.

## Supporting Information

File S1 Trial protocol.
(DOCX)

File S2 STROBE checklist.
(PDF)

File S3 VPC final model, by age group (adults and children).
(TIF)
