# Supplementary material for: Population Pharmacokinetic Study of Benznidazole in Pediatric Chagas Disease Suggests Efficacy despite Lower Plasma Concentrations than in Adults
Source: PLoS Negl Trop Dis. 2014 May 22;8(5):e2907. doi: 10.1371/journal.pntd.0002907 (PMC4031103; doi:10.1371/journal.pntd.0002907)
Supplement: File S1 — Trial protocol. (DOCX) [file pntd.0002907.s001.docx]

**POPULATION PHARMACOKINETICS STUDY OF BENZNIDAZOLE IN CHILDREN WITH CHAGAS DISEASE**

Principal Investigators:

Dr Jaime Altcheh (Children’s Hospital of Buenos Aires, Buenos Aires, Argentina)

Dr Facundo Garcia Bournissen

English version of Spanish version 2.1 – July 2007

**population pharmacokinetics Study of benznidazole in children with Chagas disease**

**Principal Investigators**

Dr. Altcheh, Jaime

Parasitology

Children’s Hospital “R. Gutiérrez”

Gallo 1330, C1425EFD, Ciudad de Buenos Aires.

Dr. Koren, Gideon

Division of Clinical Pharmacology and Toxicology

The Hospital for Sick Children, University of Toronto,

555 University Ave, 8^th^ floor, Toronto, ON M5G 1X8, Canada

**Coinvestigators**

Dr. Garcia Bournissen, Facundo

Division of Clinical Pharmacology and Toxicology

The Hospital for Sick Children, University of Toronto,

555 University Ave, 8th floor, Toronto, ON M5G1X8, Canada

Dr. Giglio, Norberto

Epidemiology

Children’s Hospital “R. Gutiérrez”

Gallo 1330, C1425EFD, Ciudad de Buenos Aires.

Dr. Della Védova, Carlos Omar

Laboratorio de Servicios a la Industria y al Sistema Científico (CONICET-CIC) Departamento de Química, Facultad de Ciencias Exactas, Universidad Nacional de La Plata.

Cno. Centenario e/ 505 y 508 Gonnet - CP 1897,

La Plata , Pcia de Buenos Aires

Dr. Alejandro Nava-Ocampo

Division of Clinical Pharmacology and Toxicology

The Hospital for Sick Children, University of Toronto,

555 University Ave, 8^th^ floor, Toronto, ON M5G 1X8, Canada

# Protocol summary

| Title | Study of population pharmacokinetics of benznidazole (BNZ) in children with Chagas disease |
| --- | --- |
| Duration | 24 months |
| Objective | To study the population pharmacokinetics of benznidazole in children with Chagas disease |
| Design | Phase I study in special populations (children). Population pharmacokinetics |
| Population | Ambulatory pediatric patients with Chagas disease |
| Sample size | Total N=50  Patients will be divided in 3 groups:   - Group 1: Patients receiving the first dose of BNZ (N=12) - Group 2: Patients in steady state of BNZ (N=26) - Group 3: Patients receiving the last dose of BNZ (N=12) |
| Selection Criteria | Children 2 to 12 years old with infection due to Trypanosome Cruzi (Chagas disease) |
| Inclusion Criteria | Children 2 to 14 year old under treatment with BNZ for Chagas disease.  Diagnosis of Chagas disease with at least 2 techniques (ELISA, hemagglutination or particle agglutination).  Informed consent provided by the parents and assentment provided by the patient. |
| Exclusion Criteria | Patients with hypersensitivity to BNZ.  Immunocompromised patients.  Altered liver or kidney function.  Pregnancy. |
| Treatment | Benznidazole 5-8 mg/kg/day p.o. in 2 daily doses for 60 days |
| Safety monitoring | Vital signs, adverse events, laboratory markers. |
| Samples for pharmacokinetics study | Samples per patient: 3 (sparse sampling)  Volume of blood per sample: 3ml |

# TABLE OF CONTENTS

1 Protocol summary 3

2 TABLE OF CONTENTS 4

3 GENERAL INFORMATION 5

3.1 Study Title 5

3.2 Principal Investigators 5

3.3 Coinvestigators 5

4 INTRODUCTION 6

4.1 Background 6

5 OBJEcTIVe 10

6 study design 10

6.1 Study type 10

6.2 Population 10

6.2.1 Selection criteria 10

6.2.2 Inclusion criteria 10

6.2.3 Exclusion Criteria 10

6.2.4 Criteria for withdrawal from the study 11

7 research plan 12

7.1 General overview of the study 12

7.2 Patient recruitment 13

7.3 Study duration 13

7.4 Data collection 13

7.5 Treatment 13

7.6 Follow up 13

7.7 Sample obtention for pharmacokinetics 14

7.7.1 Group 1: Patients receiving the first dose of benznidazole 14

7.7.2 Group 2: Patients in steady state phase (Days 3 – 59) 15

7.7.3 Group 3: Patients receiving the last dose 15

7.8 Overview of diagnostic studies 15

8 statistical design 15

8.1 Statistical methods 16

8.2 Sample size calculation 16

8.2.1 Sample size justification 16

8.2.2 Stratification of patient population 16

8.2.3 Interim analysis 16

9 ethical considerations 17

10 Adverse drug events 17

11 data confidentiality 17

12 references 18

# GENERAL INFORMATION

## Study Title

Study of population pharmacokinetics of benznidazole (BNZ) in children with chagas disease

## Principal Investigators

Dr. Koren, Gideon

Division of Clinical Pharmacology and Toxicology

The Hospital for Sick Children, University of Toronto,

555 University Ave, 8^th^ floor, Toronto, ON M5G 1X8, Canada

Dr. Altcheh, Jaime

Division of Parasitology

Hospital de Niños Ricardo Gutiérrez

Gallo 1330, C1425EFD, Ciudad de Buenos Aires.

## Coinvestigators

Dr Facundo Garcia Bournissen

Division of Clinical Pharmacology and Toxicology

The Hospital for Sick Children, Toronto, Canada.

Dr. Giglio, Norberto

Division of Epidemiology

Hospital de Niños Ricardo Gutiérrez

Gallo 1330, C1425EFD, Ciudad de Buenos Aires.

Dr. Carlos Della Vedova

Laboratorio de Servicios a la Industria y al Sistema Científico (CONICET-CIC) Departamento de Química, Facultad de Ciencias Exactas, Universidad Nacional de La Plata.

Cno. Centenario e/ 505 y 508 Gonnet - CP 1897,

La Plata , Pcia de Buenos Aires

Dr. Alejandro Nava-Ocampo

Division of Clinical Pharmacology and Toxicology

The Hospital for Sick Children, University of Toronto,

555 University Ave, 8^th^ floor, Toronto, ON M5G 1X8, Canada

# INTRODUCTION

##

## Background

Chagas disease is a parasitic infection caused by the parasite Trypanosome cruzi. The geographical distribution of this disease is wide, from the south of the USA to the south of Argentina, with endemic characteristics from the north of Mexico to the north of Argentina.^1^

The parasite that causes this disease can be transmitted by an arthropod vector, transfusions or mother to child transmission during pregnancy. Great progress has been achieved in the last years in the control of the transmission by the vector and by transfusion. Nevertheless, many challenges for the control of this infection still remain.

After the initial phase of the infection, which happens mainly in children and has a mortality of up to 10%, an asymptomatic phase ensues that will eventually lead to irreversible heart and /or intestinal tract damage in up to 30% of the patients many years after the initial infection.^1^

It is estimated that there are currently about 20 million people infected in Latin America, and that 200,000 new cases and 20,000 deaths due to complications of the disease happen every year.^1;2^ Most of the new infections happen in children, either by vector transmission or by vertical transmission. In Argentina, where 89% of the population lives in urban areas in particular in or around Buenos Aires, most of the patients infected with Chagas’ disease live in urban areas. Prevalence of Chagas’ disease in Buenos Aires has been estimated to be about 2%. Most new infections in urban areas are due to vertical transmission and occasionally due to blood transfusions. The transplacental route of transmission explains why most new cases in urban centers are detected in young children.

It has been shown that treatment of the infection in the acute phase is effective and carries a cure rate above 90%. Clinical trials have shown that the treatment of children in the quiescent phase leads to a positive therapeutic response.

New tools are being used to evaluate the response to the treatment such as PCR to detect parasite genome in blood and tissues and newer antigens for antibody tests. These advances have led to health authorities of Latin American countries and the World Health Organization to implement treatment programs of children with Chagas’ disease across central and Latin America.^3^

There are only 2 drugs currently available for the treatment of Chagas disease, nifurtimox and benznidazole.^1^

Benznidazole(N-benzyl-2-nitroimidazol-1-acetamide) is a nitroimidazol compound that was developed over 30 years ago.^4^ The mechanism of action of benznidazole is still not clear, but it has been shown to induce reductive stress leading to covalent modification of proteins and other macromolecules in the parasite.^1;5^ Another potential mechanism of action is the inhibition of the enzyme tripanotionine reductase, which leads to tripanotionine depletion, a molecule with important detoxification functions in the parasite T. cruzi.^4^ Benznidazole, and possibly its metabolites, like nifurtimox, induces the production of free radicals that can damage the parasite’s DNA, as well as inhibit RNA and protein synthesis. ^4;6^

Only 2 studies on the pharmacokinetics of benznidazole have been published to date, both in the 1970’s.

Benznidazole pharmacokinetics has been described as monocompartimental in healthy male adults.^7-9^ After a group of healthy male volunteers received 100 mg of benznidazole po, the plasmatic peak concentration of 2.2 – 2.8 mg/l was observed 3 – 4 hours post dose. The estimated half life was 12 hours, and the volume of distribution 0.56 l/kg.^8^ In a second study of multiple doses of benznidazole in Chagas patients, doses of 7 mg/kg produced plasma concentrations similar to those estimated based on the pharmacokinetic parameters obtained from the previous study.^9^ All patients reached steady state within 10 days of treatment with plasma concentrations between 5.4 – 16.4 mg/l (estimated required plasma concentration to kill the parasite: 3 – 6 mg/l).

Benznidazole is eliminated by the liver, with less than 20% excreted unchanged by the kidney. Protein binding is moderate (40%), and distribution into tissues is good, including central nervous system, liver, and red blood cells. There are virtually no studies on the metabolism of benznidazole in humans, and very little and outdated information is available from animal studies.

Benznidazole, and other drugs used for the treatment of Chagas disease have never been adequately studied in the pediatric population.^10;11^ Also, there is no appropriate formulation for use of this drug in the pediatric population (liquid formulation), which many times forces health care providers to recur to pill fractionation, a methodology fraught with difficulties and risks.^12;13^

In response to this dearth of information in the pediatric population, the Scientific Working Group on Chagas Disease, TDR, WHO/PAHO has signaled the study of the pharmacokinetics of benznidazole and nifurtimox in children as a research priority.

Pharmacokinetic studies have traditionally involved a limited number of patients, usually healthy volunteers. Pharmacokinetic profile of the studied drug would usually be characterized extensively in each individual participant, which requires a large number of samples to be obtained. The requirement for extensive sampling makes traditional pharmacokinetics studies very difficult to perform in pediatric populations due to ethical and logistical limitations (e.g. blood volume restrictions, inordinate number of punctures, etc).

In the last few decades, a new approach to pharmacokinetics, population pharmacokinetics, has gained momentum as the method of choice when studying vulnerable populations such as children. This method changes the focus away from the complete estimation of the pharmacokinetics parameters of each individual participant towards the estimation of the parameters (and its variability) in the whole population. In order to estimate the pharmacokinetics of a drug from a population perspective, a larger number of individuals is required to participate, but the amount of samples per individual is greatly reduced (to as little as 2 samples in some designs). This milder burden on the individual patient makes population pharmacokinetics the method of choice for studies in the pediatric population.

This method has been recommended by the FDA and EMEA as the standard method for pharmacokinetic studies in children.^14-16^

Given the absolute absence of data on the pharmacokinetics of benznidazole in the pediatric population, we propose to carry out a population pharmacokinetics study of this drug in children receiving this drug for the treatment of Chagas disease.

# OBJEcTIVe

To study the pharmacokinetics of benznidazole in children with Chagas’ disease.

# study design

## Study type

Population pharmacokinetics study.

Observational prospective study in especial populations (children).

## Population

### Selection criteria

Children 2–12 years old, with Chagas’ disease*.*

### Inclusion criteria

- Children 2 – 12 years old, both sexes, with a diagnosis of Chagas’ disease and eligible for treatment with benznidazole, as per current treatment protocols.
  - Chagas’ disease diagnostic criteria: At least 2 positive serological tests for *Trypanosoma cruzi* infection técnicas serológicas reactivas (ELISA, Hemmoagglutination and particle agglutination tests).
- Informed consent signed by the parents and assentment of the patient.

### Exclusion Criteria

- Patients with a history of hypersensitivity to benznidazole or any of the drug excipients.
- Immunocompromised patients (Clinical history compatible with HIV infection, cancer, primary immunodeficiencies or prolonged treatment with corticosteroids or other immunosuppressants)
- Altered hepatic function (increase in AST/ALT x3 or billirubin x3).
- Altered renal function (increase in creatinine x3).
- Pregnancy

### Criteria for withdrawal from the study

- Non-compliance with treatment plan
- Severe drug reaction potentially attributable to benznidazole
- Any situation that may put the safety of the patient at risk, according to the judgment of the researchers or attending physicians.

#

# research plan

## General overview of the study

Pretreatment period

(Days -30-0)

Treatment period (Days 1- 60)

Post-Treatment Period

(Days 61-63**)**

Clinical history, physical exam, CBC, Chagas serology, Liver and kidney function tests (pretreatment, days 0, 7, 30 and 60)

**Group 1**: N=12

Patients taking the first dose

(Sampling windows:

a) 0 - 2 hs;

b) 2 - 6 hs

c) 6 - 12 hs

post dose)

**Group 2**: N=26

Patients in steady state (Days 3 - 59)

(Sampling windows:

1. through – before next dose
2. 0 - 2 hs post dose
3. 2 - 6 hs post dose)

**Group 3**: N=12

Patients taking the last dose (Days 61 - 63)

(Sampling windows: a) 12 - 18 hs

b) 18 - 24 hs

c) 24 - 36 hs

post dose)

Safety monitoring and adverse events recording

**Day 0**

**Day 30**

**Day 60**

**Day 7**

Children 2-12yo with Chagas’ disease under treatment with benznidazole,5-8 mg/kg/d bid po

Routine visits for treatment and follow up

## Patient recruitment

Patients eligible for the study will be recruited at the Parasitology Division, Children’s Hospital of Buenos Aires, Buenos Aires, Argentina, where these patients are routinely treated and followed up. Recruitment will be carried out by the treating pediatrician and principal investigator, Dr. Jaime Altcheh.

## Study duration

The recruitment phase of the study will take an estimated 24 months.

## Data collection

Information on clinical examination, weight, height, cardiological evaluation, and clinical biochemistry results will be collected. These studies are done routinely in children with Chagas’ disease under treatment with benznidazole.

## Treatment

The patients will receive, as per routine treatment protocol of Chagas disease, Benznidazole (RADANIL®, Roche). Dose: 5-8 mg/kg/d, bid, PO for 60 days. The patients are usually treated in an outpatient setting.

Benznidazole will be provided by the Argentine National Ministry of Health, for free. The patients will be given enough benznidazole for 1 month of treatment plus 5 days. Pill counts will be carried out at the day 30 visit, when enough benznidazole to complete the remaining of the treatment will be given to the patient’s family. Pill counts will be carried out again at the end of the treatment period.

The patients parents will be given a treatment diary to complete during the treatment period with daily times of ingestion of the medication and any events that they think might be related to the treatment.

All concomitant medications will be registered in the patient’s chart. There is no available information to date neither on the metabolism of benznidazole, nor on potential drug interactions.

## Follow up

Patients will be followed according to the usual management protocol of children with Chagas’ disease, at the ambulatory clinic of the Division of Parasitology, Children’s Hospital of Buenos Aires.

Patients will be seen at the clinic at diagnosis, 7, 30 and 60 days after the start of the treatment. Clinical examination and laboratory studies will be performed at each visit, including CBC, liver and renal function tests and Chagas serology.

Parents will be instructed to report adverse events potentially related to the treatment.

Patients will be seen 3 months after the treatment period is completed, and every 6 months after that. Serology for Chagas’ disease will be performed at each visit.

All adverse events will be immediately reported to the pharmacovigilance division of the ANMAT (Argentine National Administration of Medications and Medical Technology).

## Sample obtention for pharmacokinetics

The patients enrolled in the study will be divided in 3 groups:

- Group 1: Patients receiving the first dose of benznidazole (N=12; Sampling windows: a=0-2hs; b=2-6hs; c=6-12hs post dose).
- Group 2: Patients in steady state phase (at least after 3 days of treatment; Days 3-59).(N=26; sampling windows: a=through–before next dose; b=0-2hs; c=2-6hs post dose).
- Group 3: Patients receiving the last dose of benznidazole (Days 61-63).(N=12; Sampling windows: a=12-18hs; b=18-24hs; c=24-36hs post dose).

Time of drug administration and blood extraction will be registered in a form. Extraction times will be distributed across the sampling windows (see 7.1), and taking into account, to the extent possible, patient availability and preferences.

Blood extraction will done using a butterfly needle, with previous application of anaesthetic cream. Blood extractions will be done, whenever possible, within the context of other routine blood extractions required for the clinical follow up of the patient.

Samples will be stored in the fridge until extraction (less than 48 hs).

Benznidazole extraction and measurement: Benznidazole will be extracted and measured (by HPLC) according to published protocols.^17-21^

### Group 1: Patients receiving the first dose of benznidazole

Number of patients: 12.

Number of blood extractions per patient: 3. The first extraction between 0 and 2 hours, the second extraction between 2 and 6 hours and the third extraction between 6 and 12 hours post dose.

Blood volume per extraction: 3ml.

Minimal interval between samples: 1 hour.

### Group 2: Patients in steady state phase (Days 3 – 59)

Number of patients: 26

Number of blood extractions per patient: 3. The first extraction at through (right before the next dose), the second extraction between 0 and 2 and the third extraction between 2 and 6 hours post dose.

Blood volume per extraction: 3ml.

Minimal interval between samples: 1 hour.

### Group 3: Patients receiving the last dose

Number of patients: 12

Number of blood extractions per patient: 3. Extractions times will be chosen from the following times: 12, 18, 24 and 36 hours post dose.

Blood volume per extraction: 3ml.

Minimal interval between samples: 1 hour.

## Overview of diagnostic studies

| Visit | 0 | 1 | 2 | 3 | 4 |
| --- | --- | --- | --- | --- | --- |
| Time | 0 | 7 d | 1m | 2m | posttreatment |
| Informed consent | X |  |  |  |  |
| Clinical history | X | X | X | X | X |
| Physical exam | X | X | X | X | X |
| Chagas’ serology | X | X | X | X | X |
| CBC | X | X | X | X |  |
| Liver enzymes | X | X | X | X |  |
| Creatinine | X | X | X | X |  |
| Cardiological exam | X |  |  |  |  |
| Adverse event evaluation |  | X | X | X | X |
| Drug measurements (may not coincide with clinical visits) |  | X | X | X | X |

# statistical design

## Statistical methods

Descriptive statistics (i.e. mean, median, sd, variation coefficient, etc) will be used to describe the analyzed variables.

The pharmacokinetics parameters to be estimated in the population will be Volume of distribution (V), clearance (C), absorption rate constant (Ka/F), elimination rate constant (Ke).

The population pharmacokinetic model most appropriated for the data will be chosen using the Akaike information criteria. Pharmacokinetic data will be analyzed using the program NONMEM for nonlinear mixed effects modelling.

## Sample size calculation

Fifty patients will be enrolled in the study.

### Sample size justification

For a drug following one-compartment kinetics, as has been suggested for benznidazole ^8;9^, between 10 and 20 patients would be required to detect a subpopulation with a 30% difference in drug clearance.

Given the fact that there is virtually no data available on the disposition of benznidazole in children (and very little in adults, too), we assume that 50 patients should be enough to detect the existence of 2 subpopulations and account for patient attrition.

### Stratification of patient population

Samples obtained from patients in the first group (Patients receiving the first dose of benznidazole) will provide information mainly on the absorption rate constant and volume of distribution of the drug. Samples from the remaining two groups (Patients in steady state phase and patients receiving the last dose) will provide information mainly on the clearance rate of the drug, as well as confirming if the drug follows a one-compartment model (or other).

### Interim analysis

We expect to carry out an interim analysis when 50% of the patients are enrolled. This will allow us to adjust the number of patients required and better estimate the time required to complete the study.

# ethical considerations

Informed written consent will be required of the patients’ parents or guardians, and of the patients that are capable of understanding the written consent process. Patients who cannot understand the written consent process will be required to assent, and the assent will be registered by a witness.

The study will be done in agreement with the guidelines of the International Conference on Harmonisation (ICH6), Good clinical practice guidelines (GCP), the declaration of Helsinki, the UNICEF convention on the rights of children, and the regulations of the ANMAT (Argentine Ministry of Health).

# Adverse drug events

Every adverse event, whether related to benznidazole or not, will be registered and notified to the pharmacovigilance division of the ANMAT, as well as the Teaching and Research Committee of the Children’s Hospital of Buenos Aires.

# data confidentiality

Patient data confidentiality will be strictly enforced, including patient anonymity. Samples will be identified by alphanumeric code, and patient data will be stored in files and computer to which the principal investigator and associate investigators only will have access.

# references

(1) Urbina JA, Docampo R. Specific chemotherapy of Chagas disease: controversies and advances. Trends Parasitol 2003; 19(11):495-501.

(2) WHO Expert Committee on the Control of Chagas Disease, World Health Organization. Control of Chagas disease second report of the WHO Expert Committee. 905 ed. Geneva: World Health Organization, 2002

(3) Control of Chagas disease second report of the WHO Expert Committee. WHO Expert Committee on the Control of Chagas Disease. 2002. Geneva, World Health Organization.

(4) Raether W, Hanel H. Nitroheterocyclic drugs with broad spectrum activity. Parasitol Res 2003; 90 Supp 1:S19-S39.

(5) Moreno SN, Docampo R, Mason RP et al. Different behaviors of benznidazole as free radical generator with mammalian and Trypanosoma cruzi microsomal preparations. Arch Biochem Biophys 1982; 218(2):585-591.

(6) Docampo R. Sensitivity of parasites to free radical damage by antiparasitic drugs. Chem Biol Interact 1990; 73(1):1-27.

(7) Lau AH, Lam NP, Piscitelli SC et al. Clinical pharmacokinetics of metronidazole and other nitroimidazole anti-infectives. Clin Pharmacokinet 1992; 23(5):328-364.

(8) Raaflaub J, Ziegler WH. Single-dose pharmacokinetics of the trypanosomicide benznidazole in man. Arzneimittelforschung 1979; 29(10):1611-1614.

(9) Raaflaub J. Multiple-dose kinetics of the trypanosomicide benznidazole in man. Arzneimittelforschung 1980; 30(12):2192-2194.

(10) The Therapeutic Orphan--30 Years Later. Proceedings of a joint conference of the Pediatric Pharmacology Research Unit Network, the European Society of Developmental Pharmacology, and the National Institute of Child Health and Human Development. Washington DC, USA, May 2, 1997. Pediatrics 1999; 104(3 Pt 2):581-645.

(11) Wilson JT. An update on the therapeutic orphan. Pediatrics 1999; 104(3 Pt 2):585-590.

(12) Schechter NL, Berde CB, Yaster M. Pain in infants, children, and adolescents. 2nd ed ed. Philadelphia: Lippincott Williams & Wilkins, 2002

(13) Nahata MC. Lack of pediatric drug formulations. Pediatrics 1999; 104(3 Pt 2):607-609.

(14) Meibohm B, Laer S, Panetta JC et al. Population pharmacokinetic studies in pediatrics: issues in design and analysis. AAPS J 2005; 7(2):E475-E487.

(15) Roy A, Ette EI. A pragmatic approach to the design of population pharmacokinetic studies. AAPS J 2005; 7(2):E408-E420.

(16) Sheiner LB, Beal SL. Evaluation of methods for estimating population pharmacokinetics parameters. I. Michaelis-Menten model: routine clinical pharmacokinetic data. J Pharmacokinet Biopharm 1980; 8(6):553-571.

(17) Workman P, White RA, Walton MI et al. Preclinical pharmacokinetics of benznidazole. Br J Cancer 1984; 50(3):291-303.

(18) Workman P, Walton MI, Lee FY. Benznidazole: nitroreduction and inhibition of cytochrome P-450 in chemosensitization of tumour response to cytotoxic drugs. Biochem Pharmacol 1986; 35(1):117-119.

(19) Morilla MJ, Prieto MJ, Romero EL. Benznidazole vs benznidazole in multilamellar liposomes: how different they interact with blood components? Mem Inst Oswaldo Cruz 2005; 100(2):213-219.

(20) Morilla MJ, Benavidez PE, Lopez MO et al. Liposomal benznidazole: a high-performance liquid chromatographic determination for biodistribution studies. J Chromatogr Sci 2003; 41(8):405-409.

(21) de Castro CR, az de Toranzo EG, Castro JA. Benznidazole-induced ultrastructural alterations in rat adrenal cortex. Mechanistic studies. Toxicology 1992; 74(2-3):223-232.
