# Supplementary figures and images for: Population Pharmacokinetic Study of Benznidazole in Pediatric Chagas Disease Suggests Efficacy despite Lower Plasma Concentrations than in Adults
Source: PLoS Negl Trop Dis. 2014 May 22;8(5):e2907. doi: 10.1371/journal.pntd.0002907 (PMC4031103; doi:10.1371/journal.pntd.0002907)

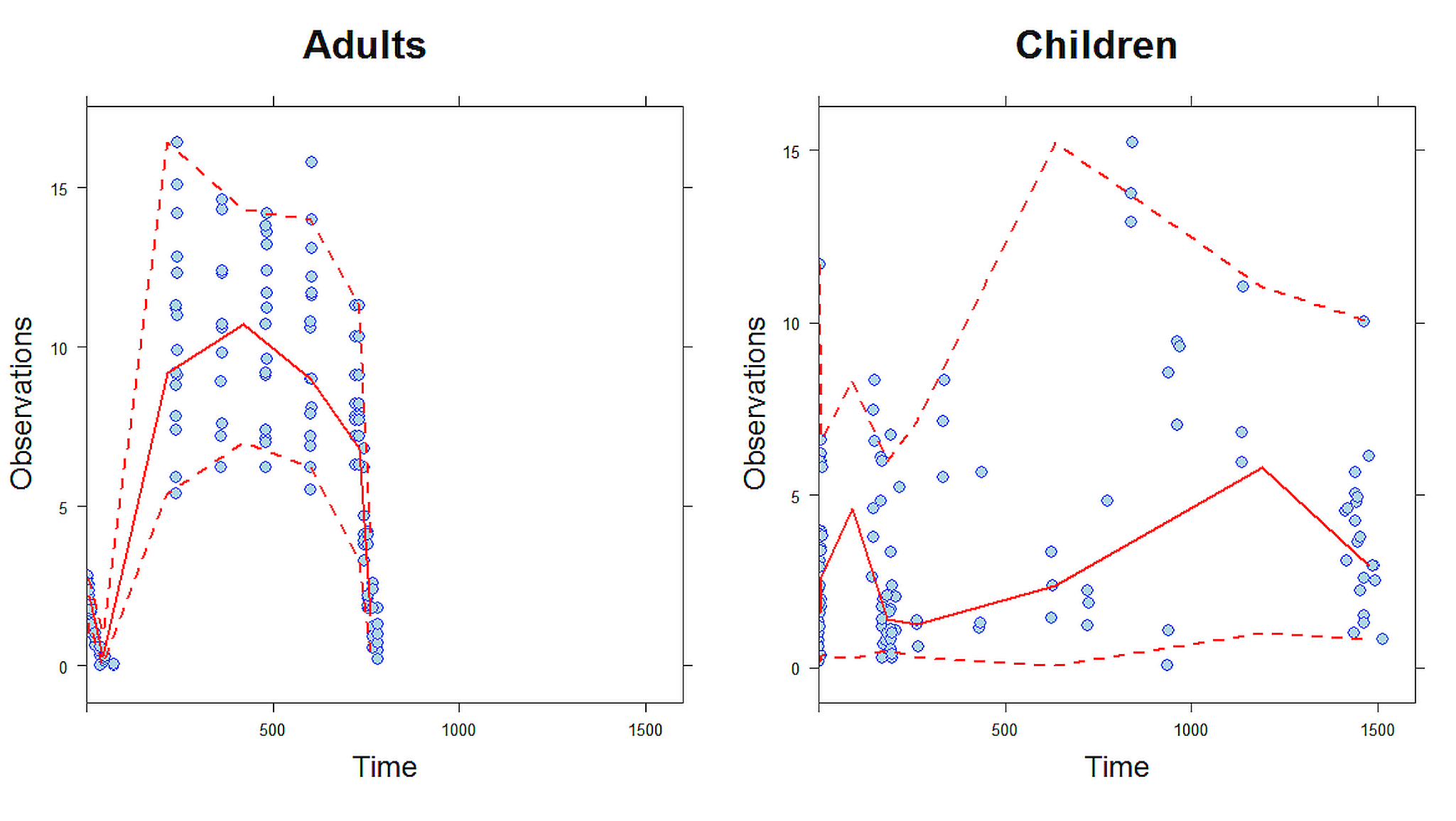

Supplement: File S3 — VPC final model, by age group (adults and children). (TIF) [file pntd.0002907.s003.tif]
